# Impact of Hydrogel-Coated Chest Drains on Outcomes in Thoracic Surgery

**DOI:** 10.1093/icvts/ivaf290

**Published:** 2025-12-01

**Authors:** Akshay J Patel, Stefano Cafarotti, Thomas Kiefer, Francesco Leo, Puiyee Sophia Chan, Federico Femia, Adele Tessitore, Miriam Patella, Simona Sobrero, Andrea Bille

**Affiliations:** Division of Thoracic Surgery, Toronto General Hospital, Toronto, ON M5G 2C4, Canada; Institute of Immunology and Immunotherapy, University of Birmingham, Edgbaston, Birmingham B15 2TT, United Kingdom; Thoracic Surgery Department, Ente Ospedaliero Cantonale, Bellinzona, Switzerland; Thoracic Surgery Department, Lungezentrum Bodensee, Konstanz, Germany; Thoracic Surgery Department, Ospedale S. Luigi Gonzaga, Orbassano, Italy; Department of Oncology, University of Turin, Turin, Italy; Department of Thoracic Surgery, Guy’s Hospital, Guy’s and St Thomas NHS Foundation Trust, London SE1 9RT, United Kingdom; Department of Thoracic Surgery, Guy’s Hospital, Guy’s and St Thomas NHS Foundation Trust, London SE1 9RT, United Kingdom; Thoracic Surgery Department, Ente Ospedaliero Cantonale, Bellinzona, Switzerland; Thoracic Surgery Department, Ente Ospedaliero Cantonale, Bellinzona, Switzerland; Thoracic Surgery Department, Ospedale S. Luigi Gonzaga, Orbassano, Italy; Department of Thoracic Surgery, Guy’s Hospital, Guy’s and St Thomas NHS Foundation Trust, London SE1 9RT, United Kingdom

**Keywords:** thoracic surgery, chest drains, hydrogel coating, postoperative complications, pleural effusion, propensity score matching

## Abstract

**Objectives:**

To compare postoperative outcomes between hydrogel-coated chest drains (HCDs) and conventional non-coated drains (NCDs) in patients undergoing general thoracic surgery, using a propensity score-matched analysis.

**Methods:**

This retrospective multi-institutional study included adult patients who underwent thoracic surgery across 4 European centres between February and September 2022. Patients were grouped according to drain type (HCD vs NCD), and a propensity score-matched analysis was performed to account for 16 preoperative and intraoperative covariates. The primary outcome was length of postoperative hospital stay (LOS). Secondary outcomes included in-hospital complications, intensive care unit (ICU) admission, chest drain reinsertion, readmission, duration of drainage, and in-hospital mortality. Subgroup analysis was performed in patients undergoing anatomical lung resections.

**Results:**

A total of 773 patients were included (HCD *n* = 362; NCD *n* = 411). After matching, 724 patients were analysed. HCD use was associated with a significantly shorter LOS (average treatment effect of the treated population −1.87 days; 95% CI −3.04 to −0.695; *P *= .002), lower odds of ICU admission (odds ratio [OR] 0.29; 95% CI 0.16-0.53; *P *< .001), and lower in-hospital complication rates (OR 0.38; 95% CI 0.26-0.55; *P *< .001). Rates of pneumonia (5.2% vs 13.4%; *P *= .001), atrial fibrillation (2.2% vs 9.0%; *P *< .001), and retained pleural effusion (0.8% vs 3.6%; *P *= .015) were significantly lower in the HCD group. There were no significant differences in drain duration, readmission, or mortality. In the anatomical resection subgroup, HCDs were similarly associated with reduced LOS and complications.

**Conclusions:**

Hydrogel-coated drains are associated with fewer postoperative complications and shorter hospital stay compared to conventional drains, particularly in anatomical lung resections. These findings support further prospective evaluation to define the role of HCDs in routine thoracic surgical practice.

## Graphical abstract

**Figure:**
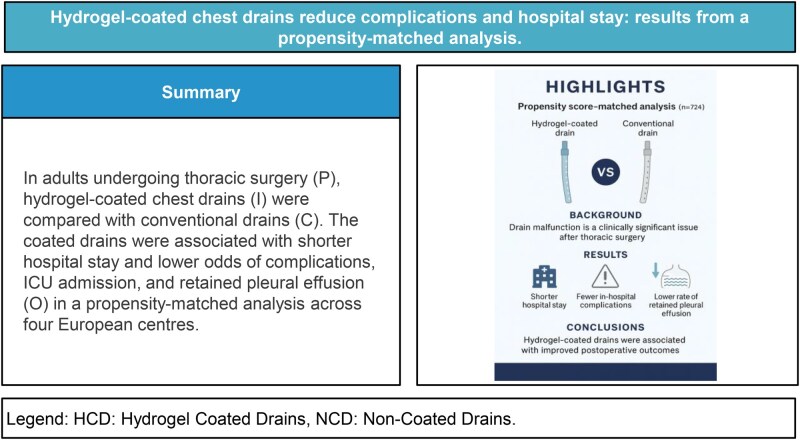

## INTRODUCTION

Chest drains are an essential component of postoperative care following thoracic surgery, facilitating evacuation of air and fluid from the pleural space to restore lung function and reduce the risk of complications such as infection, atelectasis, and pleural adhesions.^1^ However, chest drain malfunction primarily due to obstruction remains a clinically significant issue, with blockage rates reported as high as 7.4% in standard drains.^1^ Obstructed drainage can result in retained effusions, empyema, subcutaneous emphysema, and in some cases, necessitate reinsertion of a chest tube to manage unresolved pneumothorax or effusion.

A key determinant of drain performance is the material and surface properties of the device. Hydrogel-coated drains, by reducing the coefficient of friction, may resist clot adhesion and maintain patency more effectively than conventional drains.^2^ In cardiac surgery, hydrogel-coated drains have demonstrated significantly higher clot-free removal rates (87.4%) compared to standard (49.7%) and active clearance drains (33.3%).^3^

This study aimed to compare postoperative outcomes between patients receiving hydrogel-coated chest drains (HCDs) and those with conventional, non-coated drains (NCD) following thoracic surgery. We hypothesized that HCDs would be associated with lower rates of drain-related complications and shorter hospital stay.

## METHODS

This retrospective, multi-institutional study was conducted across 4 European general thoracic surgery centres (GH, EOC, LB, and OSLG) between February and September 2022. The study included adult patients (≥18 years) who underwent thoracic surgery involving the placement of HCDs (ClotStop Catheter; Medela AG, Switzerland). Eligible procedures encompassed anatomical lung resections for malignant or benign conditions, wedge resections, lymph node biopsies, pleural biopsies with or without pleurodesis, bullectomy and pleurodesis for primary or secondary pneumothorax, empyema washouts, and pleurectomy with or without decortication for mesothelioma or benign disease. Patients who were sent home with a chest drain in situ were excluded from the analysis.

Hydrogel-coated drains were introduced at each participating centre following approval by their respective medical equipment management services. Their adoption was based on both availability and preliminary evidence suggesting improved patency and reduced blockage risk, even at smaller diameters. For comparison, a historical control cohort of patients who underwent thoracic surgery during the 8 months preceding the introduction of hydrogel-coated drains was retrospectively identified.

Patients underwent thoracic procedures via open, video-assisted thoracoscopic (VATS), or robotic-assisted thoracic surgery (RATS) approaches, determined by clinical indication, surgeon experience, and institutional practice. Chest drain size (20-28 Fr) was selected according to procedure type and surgeon preference. Drains were connected to either a traditional water-seal collection system or a digital suction device (Thopaz+ Digital Chest Drainage, Medela AG, Switzerland), with suction pressures ranging from −8 to −20 cmH_2_O. Perioperative management followed standard institutional protocols.

### Data collection

For all included patients, the following variables were extracted from anonymized electronic medical records: demographic data, surgical indication, operative approach (open, VATS, or RATS), and the type of procedure performed. Postoperative clinical outcomes were also recorded, including the length of hospital stay (LOS), defined as the number of inpatient days following the day of surgery (day 0), and the length of chest drain duration, defined as the number of days the patient had a drain in situ postoperatively.

The primary outcome measure was the length of postoperative stay. Secondary outcomes included the need for chest drain reinsertion, in-hospital complication rate, hospital readmission for complications such as recurrent pneumothorax or surgical emphysema, length of chest drain drainage in days, need for intensive care unit (ICU) admission and in-hospital mortality. Additionally, drain-related complications were recorded, including blocked drains resulting in retained pneumothorax or haemothorax, unexplained surgical emphysema in the absence of an air leak, and bleeding associated with drain insertion.

Patients were divided into 2 cohorts: those who received HCDs (HCD group) and those who received conventional NCDs (NCD group). Subgroup analyses were also conducted in the anatomical lung resection cohort (lobectomy, bilobectomy, or segmentectomy).

### Statistical analysis

Categorical variables were compared using the chi-square test, while continuous variables were assessed using the Mann-Whitney *U*-test. A Bonferroni-adjusted significance threshold (*P *< .05) was applied to account for multiple comparisons across primary outcomes.

Propensity score matching was used to estimate the effect of “drain type” on various postoperative parameters (continuous and binary outcome modelling). The propensity scores were estimated using logistic regression based on age, sex, performance status, presence of comorbidities (pulmonary or cardiac), the operative approach, laterality of procedure, procedure type, operative time, intraoperative blood loss, centre, drain size, number of drains placed, suction application, degree of suction applied, type of drainage system (underwater seal or digital), and analgesia protocol (epidural, paravertebral catheter, opioid PCA, simple analgesia). One-to-one nearest neighbour matching was used. Seven hundred and twenty-four patients from the original 773 patient cohort were matched according to the drain type used (HCD vs NCD). A reasonable balance was achieved between the groups, with all standardized mean differences for squares and two-way interactions between covariates were below 0.15, indicating adequate balance. Caliper matching (0.2 times the SD of the logit of the propensity score) was employed as per Austin et al.^4^ All patients within the range of propensity scores where both treated and control subjects exist were included in the analysis.

Logistic and linear regression modelling were employed according to the data distribution of the outcome variable with drain type as the exposure, along with covariates and their interaction as predictors. We included full matching weights in the estimation. The comparisons () function in the *marginaleffects* package was used to perform g-computation in the matched sample to estimate the average treatment effect of the treated population (ATT). A cluster-robust variance was used to estimate its standard error with matching stratum membership as the clustering variable. All analyses were conducted using R 4.2.3 and the *MatchIt, cobalt, sandwich, lmtest,* and *marginaleffects* packages.^5–8^

### Ethical considerations

All patient data were obtained from anonymized electronic medical records. The study was conducted in accordance with the Declaration of Helsinki and adhered to institutional data protection guidelines. Ethical approval was not required, as the project was classified as a Quality Improvement Evaluation. Informed consent was waived due to the retrospective nature of the study and the use of fully anonymized data. Stored data from research participants for multiple uses was consistent with the requirements outlined in the WMA Declaration of Taipei.

## RESULTS

A total of 773 patients undergoing thoracic surgery were included in the study, comprising 362 patients in the HCD and 411 in the conventional NCD group. Baseline characteristics are summarized in **Table 1**.

**Table 1.** A Comparison of Patient Characteristics Stratified to Drain Subtype

Strata	HCD	NCD	*P*-value
Patients (*n*)	362	411	–
Mean age (mean ± SD)	64 ± 14	64 ± 15	NS
Female sex (%)	172 (47.5)	212 (51.6)	NS
**Indication for surgery (%)**			
Benign diseases	99 (27.3)	110 (26.8)	NS
Malignant diseases	263 (72.7)	301 (73.2)	NS
**Surgical approach (%)**			
Open	55 (15.2)	89 (21.7)	.026
VATS	171 (47.2)	55 (13.4)	<.001
RATS	136 (37.6)	267 (65.0)	<.001

Abbreviations: HCD, hydrogel-coated chest drain; NCD, non-coated drain; RATS, robotic-assisted thoracic surgery; VATS, video-assisted thoracoscopic surgery.

The proportion of patients with an undefined surgical indication was significantly higher in the HCD group. Surgical approach differed between groups: a greater proportion of HCD patients underwent VATS procedures, while RATS was more frequently employed in the NCD group.

Following propensity score matching (1:1), groups were balanced across 16 baseline covariates (**Table 2**). The matched analysis demonstrated that HCDs were associated with improved postoperative outcomes in several domains. HCDs associated with a significantly shorter LOS, with an ATT of −1.87 days (−3.04 to −0.695; *P *= .002). HCDs were associated with a significantly reduced odds of ICU admission (OR 0.289; 95% CI 0.157-0.529; *P *< .001) and in-hospital complications (OR 0.378; 95% CI 0.261-0.549; *P *< .001). There was no impact of HCDs on in-hospital mortality, readmission rate or on the duration of drainage. There was no measurable pleural effusion seen on CXR prior to drain removal in any of the patients in this series. All re-admissions reported were related to the complications outlined in **Tables 3 and 4** (pleural effusion, pneumothorax, need for drain reinsertion for either of these indications). **Table 3** demonstrates a breakdown of the complication rates between groups.

**Table 2.** A Comparison of Patient Characteristics Stratified to Drain Subtype Post Propensity Matching

Strata	HCD	NCD	*P*-value
Patients (*n*)	362	362	–
Mean age (mean ± SD)	65 ± 11	64 ± 10	NS
Female sex (%)	172 (47.5)	186 (51.4)	NS
**Indication for surgery (%)**			
Benign diseases	78 (27.3)	81 (22.4)	NS
Malignant diseases	284 (72.7)	281 (77.6)	NS
**Surgical approach (%)**			
Open	55 (15.2)	52 (14.4)	NS
VATS	171 (47.2)	55 (15.2)	.039
RATS	136 (37.6)	255 (70.4)	<.001

Abbreviations: HCD, hydrogel-coated chest drain; NCD, non-coated drain; RATS, robotic-assisted thoracic surgery; VATS, video-assisted thoracoscopic surgery.

**Table 3.** Complications Stratified to Drain Type

Complication	HCD	NCD	*P*-value
Pneumonia (%)	19 (5.2)	55 (13.4)	.001
Prolonged air leak (%)	18 (5.0)	23 (5.6)	NS
Atrial fibrillation (%)	8 (2.2)	37 (9.0)	<.001
**Drain-specific complications (%)**			
Severe surgical emphysema	1 (0.3)	1 (0.2)	NS
Complication after drain removal	4 (1.1)	19 (4.6)	.005
Pleural effusion	3 (0.8)	15 (3.6)	.015
Pneumothorax	1 (0.3)	4 (1.0)	NS
Chest drain reinsertion	3 (0.8)	14 (3.4%)	.024
Readmission	3 (0.8)	4 (1.0%)	NS

Abbreviations: HCD, hydrogel-coated chest drain; NCD, non-coated drain.

**Table 4.** Complications Stratified to Drain Type in the Anatomical Lung Resection Subgroup

Complication	HCD	NCD	*P*-value
Pneumonia (%)	12 (6.8)	38 (16.2)	.003
Prolonged air leak (%)	10 (5.7)	17 (7.3)	NS
Atrial fibrillation (%)	5 (2.8)	24 (10.3)	<.001
**Drain-specific complications (%)**			
Severe surgical emphysema	0 (0.0)	1 (1.4)	NS
Complication after drain removal	4 (2.3)	10 (4.3)	NS
Pleural effusion	1 (0.6)	7 (2.9)	.035
Pneumothorax	0 (0.0)	2 (0.9)	NS
Chest drain reinsertion	0 (0.0)	3 (1.3)	.044
Readmission	2 (1.1)	2 (0.9)	NS

Abbreviations: HCD, hydrogel-coated chest drain; NCD, non-coated drain.

### Anatomical lung resection subgroup

In the subgroup of patients undergoing anatomical lung resections (lobectomy, bilobectomy, or segmentectomy), the matched analysis demonstrated that HCDs associated with a significantly shorter LOS, with an ATT of −1.85 days (−3.63 to −0.0761; *P *= .041). HCDs associated with a significantly reduced odds of ICU admission (OR 0.149; 95% CI 0.00548-0.407; *P *< .001) and in-hospital complications (OR 0.48, 95% CI 0.31-0.743; *P *= .001). There was no impact of HCDs on in-hospital mortality, readmission rate or on the duration of drainage. **Table 4** shows the complication breakdown in the anatomical lung resection subgroup.

## DISCUSSION

Effective chest drainage is critical to optimize lung re-expansion and prevent complications such as empyema and tension pneumothorax following thoracic surgery.^9^^,^^10^ Drain obstruction can necessitate invasive interventions, including reinsertion or surgical re-exploration,^11^^,^^12^ and may contribute to prolonged hospital stays and increased morbidity.

Drain design including size, material composition, and surface properties plays a key role in maintaining patency. Despite advancements, current chest drain options remain suboptimal; a survey of 108 cardiothoracic surgeons found widespread dissatisfaction with available devices.^13^ When occlusion occurs, manual techniques such as milking, stripping, or flushing are commonly employed. However, these methods have limited efficacy and may increase intrathoracic pressures, potentially exacerbating complications.^14–16^

In a randomized trial, Dango et al^17^ found increased effusion output in patients undergoing drain milking, without any reduction in morbidity or mortality, reinforcing the need for passive solutions that maintain patency. Devices offering active clearance mechanisms exist but often require regular activation and add cost.^18^

In our propensity score-matched analysis, HCDs were associated with significantly improved postoperative outcomes compared to NCDs. Patients in the HCD group demonstrated a shorter length of stay and lower odds of complications and ICU admission. Importantly, the HCD group had fewer retained effusions and lower reinsertion rates, suggesting a trend towards superior drainage efficacy. These findings are consistent with prior literature showing hydrogel-coated drains resist clot formation and obstruction.^2^^,^^3^ The reduced incidence of retained fluid may reflect better continuous drainage, potentially mitigating pleural inflammation and the risk of infection. With regard to the reduced incidence of ICU admission in the HCD group, the mechanism of this is not entirely clear but perhaps relates to the reduced incidence of postoperative and indeed post-drain removal complications. In cardiac surgery, more effective drainage of retained blood and effusions with digital systems is associated with a reduced incidence of postoperative atrial fibrillation and the associated morbidity of a prolonged ICU stay.^19^^,^^20^ This all would need corroborating in a larger, prospective randomized setting.

While rates of subcutaneous emphysema and post-drain pneumothorax were not significantly different between groups, this may reflect that such complications are multifactorial and not solely dependent on drain design.^21^^,^^22^ Although improper placement is a known contributor to subcutaneous emphysema, especially in trauma settings, this is less likely in intraoperatively placed drains.^22^ Nonetheless, poor drainage due to mechanical blockage remains a modifiable risk factor.^22^

The reduced rate of reinsertion in the HCD group may partly explain the observed shorter hospital stays, though this association may be influenced by additional confounding factors such as case complexity, surgical urgency, and operator variability. These findings support the use of hydrogel-coated drains to reduce postoperative morbidity and resource utilization.

Future prospective, randomized trials are warranted to validate these findings, quantify cost-effectiveness, and assess long-term outcomes. Additionally, mechanistic studies examining clot adherence and drain patency *in vivo* would further elucidate the benefits of hydrogel coatings.

### Limitations

Despite these promising results, this study has several limitations. First, as a retrospective, multicentre analysis, it is inherently susceptible to selection bias and variation in clinical practice. Notably, chest tube management protocol including drain size, removal criteria, and insertion technique was not standardized across the participating centres. These uncontrolled variables could confound the observed associations, limiting our ability to isolate the effect of the hydrogel coating on patient outcomes. Second, although we employed propensity score matching to reduce the impact of confounding factors, we did not conduct subgroup analyses stratified by all specific surgical procedures or underlying pathologies, which may affect effusion formation and drain performance. For example, the physiologic drainage requirements after a VATS wedge resection differ substantially from those following decortication or lobectomy, and such heterogeneity may have influenced our outcomes and undermined the validity of the results. Moreover, we did not systematically measure or quantify the volume of pleural effusion and thus cannot directly link hydrogel coating to reduced effusion burden despite our observed lower reinsertion rates and complication profiles. While the indication for surgery was captured, the lack of stratified reporting or exclusion of patients with undefined operative intent may introduce additional bias. Importantly, pain, an essential postoperative outcome known to be influenced by chest tube characteristics, was not formally evaluated with pain scores in this study, although documented analgesia regimens were documented and matched between groups. Given its potential relationship with recovery, respiratory complications, and hospital stay, this omission represents a significant limitation. Ultimately, although our findings suggest a potential benefit of hydrogel-coated drains, only a prospective randomized controlled trial, with standardized protocols, stringent patient selection, focused subgroup analyses and prespecified outcomes including pain, would be capable of drawing definitive conclusions regarding their clinical utility.

## CONCLUSION

Hydrogel-coated chest drains were associated with a lower incidence of drain-related complications, particularly retained effusions and need for reinsertion, as well as shorter hospital stay. These results suggest a potential role for coated drains in optimizing postoperative care and reducing healthcare burden in thoracic surgery.

## Data Availability

The data underlying this article will be shared on reasonable request to the corresponding author.
